# Supplementary material for: A Study of Gene Expression Changes in Human Spinal and Oculomotor Neurons; Identifying Potential Links to Sporadic ALS
Source: Genes (Basel). 2020 Apr 20;11(4):448. doi: 10.3390/genes11040448 (PMC7230244; doi:10.3390/genes11040448)
Supplement: Supplementary file 1 [file genes-11-00448-s001.pdf]

Supplementary Table 1. Differentially expressed genes between healthy- and ALS-spinal tissue. Over expressed genes are in blue. Under expressed genes are in Red.

	ID	P.Value	t	logFC	Gene.symbol
1	AF002224 at	3.7200e-08	21.70	6.48	UBE3A
2	X64072 s at	3.4700e-05	8.56	6.45	ITGB2
3	M94345 at	3.3600e-03	4.18	6.04	CAPG
4	D29642 at	5.1900e-05	8.08	6.03	ARHGAP25
5	U50327 s at	6.0300e-03	3.76	5.78	PRKCSH
6	M25280 at	1.3900e-06	13.40	5.68	SELL
7	D10495 at	1.5400e-03	4.79	5.64	PRKCD
8	X93996 rna1 at	1.4800e-02	3.13	5.63	FOXO4
9	X93510 at	2.3400e-03	4.46	5.46	PDLIM4
10	M21186 at	9.8800e-03	3.41	5.45	CYBA
11	X84213 s at	1.6000e-06	13.10	5.35	BAK1
12	L21993 at	8.4300e-03	3.52	5.33	ADCY2
13	M95178 at	6.9500e-03	3.65	5.32	ACTN1
14	X52896 s at	3.1200e-04	6.19	5.29	ELN
15	X13334 at	1.0300e-02	3.38	5.29	CD14
16	S76942 s at	4.3800e-03	3.99	5.11	DRD4
17	AF008445 at	4.0800e-03	4.04	5.09	PLSCR1
18	X17094 at	4.5100e-05	8.24	5.08	FURIN
19	Y07604 at	4.5000e-03	3.97	5.00	NME4
20	U77643 at	1.1200e-02	3.32	4.99	SECTM1
21	D38555 at	4.7100e-03	3.94	4.94	SEC24C
22	D49493 at	3.8900e-04	5.98	4.93	GDF10
23	D85939 at	7.7800e-03	3.57	4.90	CFDP1
24	X12791 at	1.9400e-02	2.95	4.87	SRP19
25	L36463 at	4.3300e-03	4.00	4.84	RIN1
25	U17566 at	9.0500e-03	-3.47	-3.93	SLC19A1
24	X07618 s at	2.6500e-02	-2.74	-3.96	LOC101929829///CYP2D6///CYP2D7
23	M36200 at	1.7100e-02	-3.03	-3.97	VAMP1
22	D00003 s at	8.0400e-03	-3.55	-3.99	CYP3A4
21	L13720 at	3.9300e-02	-2.48	-4.00	GAS6
20	L11701 s at	6.6500e-03	-3.69	-4.01	GPLD1
19	X97058 at	9.7000e-03	-3.42	-4.06	P2RY6
18	X77307 at	8.8000e-04	-5.26	-4.08	HTR2B
17	U82671 cds2 at	9.6200e-03	-3.43	-4.08	ZNF185
16	U63743 at	1.2400e-02	-3.25	-4.12	KIF2C
15	D45399 at	1.0200e-02	-3.38	-4.13	PDE6H
14	X90908 at	9.6000e-05	-7.39	-4.24	FABP6
13	U82818 at	7.9300e-03	-3.56	-4.24	UCP3
12	M82962 at	1.3000e-04	-7.07	-4.25	MEP1A
11	L40586 at	3.8800e-04	-5.99	-4.27	IDS
10	U49837 at	1.3000e-02	-3.22	-4.28	CSRP3
9	L07592 at	8.1000e-03	-3.55	-4.34	PPARD
8	U37219 at	2.0600e-02	-2.91	-4.38	PPIL2
7	S79781 at	1.0700e-02	-3.35	-4.44	WT1-AS
6	U13220 at	4.3400e-02	-2.42	-4.56	FOXF2
5	U78793 at	2.9600e-03	-4.28	-4.81	FOLR1
4	M64231 rna1 at	7.9300e-03	-3.56	-5.01	SRM
3	Z73677 at	2.5400e-06	-12.30	-5.13	PKP1
2	U20530 at	2.0600e-05	-9.22	-5.35	SPP2
1	U66559 at	1.3600e-03	-4.90	-5.88	ALK

Supplementary Table 2. Differentially expressed genes between oculomotor and spinal tissue from healthy subjects. Over expressed genes are in blue. Under expressed genes are in Red.

	ID	P.Value	t	logFC	Gene.symbol
1	205311_at	3.7200e-08	9.16	7.91	DDC
2	1555332_at	3.4700e-05	3.30	6.67	TPH2
3	241811_x_at	3.3600e-03	3.96	5.92	SLC6A4
4	230883_at	5.1900e-05	5.63	5.81	NXPH2
5	219106_s_at	6.0300e-03	5.05	5.77	KLHL41
6	240713_s_at	1.3900e-06	6.56	5.71	LINC00403
7	242009_at	1.5400e-03	2.98	5.65	SLC6A4
8	209602_s_at	1.4800e-02	2.69	5.63	GATA3
9	223810_at	2.3400e-03	4.70	5.62	KLHL1
10	205857_at	9.8800e-03	6.88	5.45	SLC18A2
11	209603_at	1.6000e-06	5.21	5.35	GATA3
12	206326_at	8.4300e-03	5.38	5.20	GRP
13	229147_at	6.9500e-03	4.76	5.19	RASSF6
14	241672_at	3.1200e-04	5.95	4.95	SERTM1
15	209560_s_at	1.0300e-02	4.04	4.84	DLK1
16	230896_at	4.3800e-03	5.37	4.77	BEND4
17	227764_at	4.0800e-03	5.22	4.75	LYPD6
18	204224_s_at	4.5100e-05	5.60	4.59	GCH1
19	222108_at	4.5000e-03	4.91	4.55	AMIGO2
20	231397_at	1.1200e-02	3.91	4.46	PLPPR5
21	242524_at	4.7100e-03	4.72	4.37	CBLN4
22	207060_at	3.8900e-04	5.98	4.30	EN2
23	219937_at	7.7800e-03	3.93	4.27	TRHDE
24	210729_at	1.9400e-02	3.41	4.27	NPY2R
25	243681_at	4.3300e-03	3.55	4.23	SHANK2
25	218035_s_at	9.0500e-03	-4.41	-4.49	RBM47
24	236203_at	2.6500e-02	-5.49	-4.50	LOC100509457///HLA-DQA1
23	233760_at	1.7100e-02	-6.82	-4.51	UTP11
22	227744_s_at	8.0400e-03	-6.91	-4.56	HNRNPD
21	203477_at	3.9300e-02	-5.41	-4.60	COL15A1
20	220518_at	6.6500e-03	-5.61	-4.60	ABI3BP
19	205522_at	9.7000e-03	-4.00	-4.62	HOXD4
18	226582_at	8.8000e-04	-4.07	-4.63	LOC400043
17	1559839_at	9.6200e-03	-7.12	-4.72	TBX18
16	1570372_at	1.2400e-02	-7.68	-4.76	LOC101928207
15	229839_at	1.0200e-02	-5.16	-4.81	SCARA5
14	209795_at	9.6000e-05	-3.62	-4.84	CD69
13	209687_at	7.9300e-03	-4.71	-4.94	CXCL12
12	229604_at	1.3000e-04	-4.15	-4.96	CMAHP
11	241612_at	3.8800e-04	-4.68	-4.98	FOXD3
10	211161_s_at	1.3000e-02	-4.93	-5.03	COL3A1
9	209612_s_at	8.1000e-03	-4.36	-5.12	ADH1B
8	213764_s_at	2.0600e-02	-7.93	-5.36	MFAP5
7	1556499_s_at	1.0700e-02	-3.74	-5.39	COL1A1
6	206858_s_at	4.3400e-02	-10.35	-5.42	HOXC6
5	222722_at	2.9600e-03	-4.86	-5.46	OGN
4	218730_s_at	7.9300e-03	-9.60	-5.50	OGN
3	214651_s_at	2.5400e-06	-4.52	-5.55	HOXA10-HOXA9///MIR196B///HOXA9
2	216834_at	2.0600e-05	-4.65	-5.95	RGS1
1	238756_at	1.3600e-03	-7.19	-6.31	GAS2L3

Supplementary Figure 1. STRING-interactome network showing pathways related to identified genes.

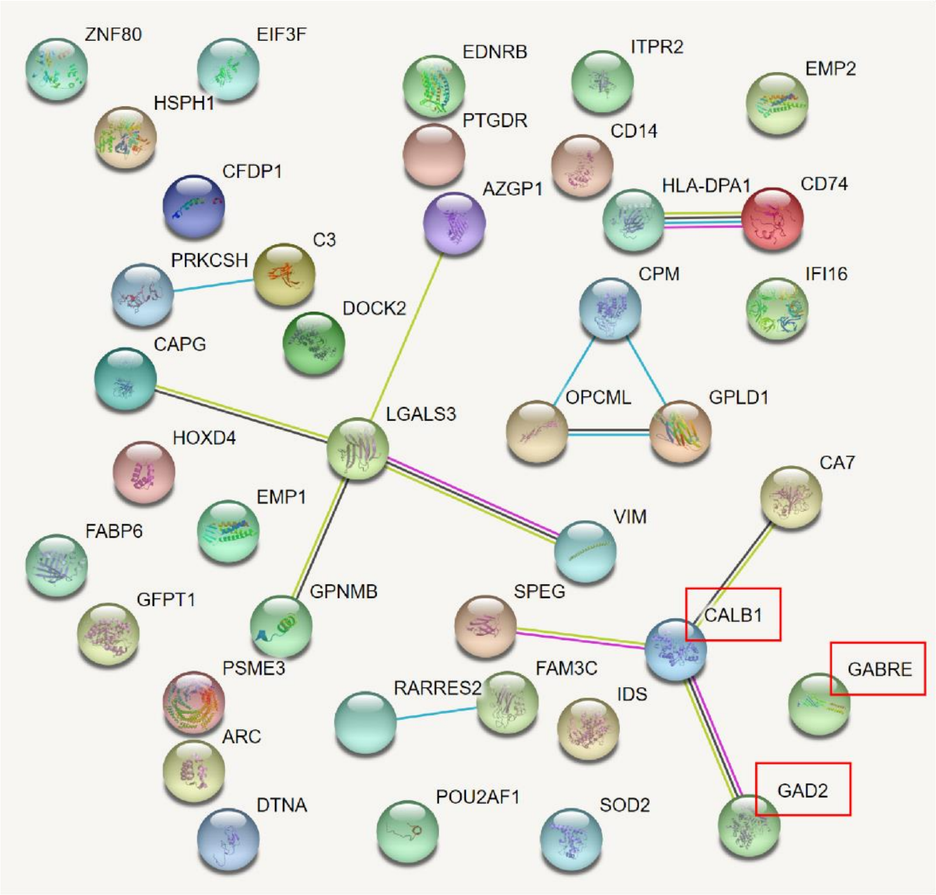

Supplementary Figure 1: Interactions between common, differentially expressed genes. STRING protein-protein interaction network consisting of both direct and indirect interactions among the 39 common, differentially expressed genes across GSE833 and GSE40438. GAD2, CALB1, and GABRE are highlighted for the significance in ALS-related pathways.
